# Colorectal cancer survivors: an investigation of symptom burden and influencing factors

**DOI:** 10.1186/s12885-018-4923-3

**Published:** 2018-10-22

**Authors:** Claire O’Gorman, Jim Stack, Alan O’Ceilleachair, Suzanne Denieffe, Martina Gooney, Martina McKnight, Linda Sharp

**Affiliations:** 1 Waterford Institute of Technology, Cork Road, Co. Waterford, Ireland; 2 National Cancer Registry, Airport Road, Co. Cork, Ireland; 3 Newcastle University, Newcastle, Upon Tyne UK

**Keywords:** Survivorship, Colorectal Cancer, Symptoms

## Abstract

**Background:**

Colorectal cancer is a significant issue internationally, with over 1.3 million people diagnosed annually. Survival rates are increasing as treatments improve, although physical symptoms can persist despite eradication of the tumour. In order to optimize survivorship care, further research is warranted in relation to symptom burden. Therefore, the objectives of this study are to (i) investigate frequency of physical symptoms in colorectal cancer survivors (ii) identify which symptoms occur together (iii) examine the associations between demographic and clinical variables, and symptoms.

**Methods:**

Participants nine months to three years post diagnosis were identified from the population-based National Cancer Registry Ireland. Respondents completed the EORTC QLQ-C30 and EORTC QLQ-CR29. Reported physical symptom frequencies were transformed into continuous scale variables, which were then analysed using one way analysis of variance, general linear modelling and Spearman rank correlations.

**Results:**

There were 496 participants. Fatigue, insomnia and flatulence were the most frequent symptoms, with ≥20% of respondents reporting these to be often present in the previous week. Eight other symptoms were experienced often by 10–20% of respondents. At least one of these eleven most common symptoms was experienced frequently by almost every respondent (99%). 66% of respondents experienced at least two of these symptoms together, and 16% experienced five or more together. Current stoma was the single most common variable associated with increased symptom scores, although statistically significant relationships (*p* ≤ 0.05) between symptom frequency scores and clinical/demographic variables were generally weak (R-sq value ≤0.08).

**Conclusion:**

Findings may inform targeted interventions during the nine month to three year post diagnosis timeframe, which would enable supported self-management of symptoms.

**Electronic supplementary material:**

The online version of this article (10.1186/s12885-018-4923-3) contains supplementary material, which is available to authorized users.

## Background

Colorectal cancer is a very significant issue worldwide with over 1.3 million people diagnosed annually [1]. However, developments in detection and treatments have led to improving survival rates, with five year survivorship increasing by 2.7% each year between 2007 and 2013 in the United States, and in the United Kingdom, rates of survival have more than doubled in the last 40 years [2, 3]_._ These trends suggest the colorectal cancer survivor population will continue to grow [4, 5]. The domino effect of this is increased survivorship burden, as current research indicates that physical symptoms can persist despite eradication of the tumour [6–15].

In the first year post completion of treatment, survivors may experience multiple moderate to severe physical symptoms [6–8]. These include diarrhoea, flatulence, frequent defecation, urinary frequency, abdominal pain, nausea and vomiting and fatigue [6–11]. While many of these symptoms improve somewhat one year post treatment [9], some can continue into longer term survivorship with bowel issues and fatigue being identified as common persistent symptoms [12–14]. In terms of symptoms and other potentially influencing factors, evidence is limited and somewhat contradictory. For example, in 2016, Foster et al. [7] found that site had little effect on symptoms up to two years after treatment whereas both Di Fabio et al. (2008) and Knowles et al. (2013) reported bowel symptoms to be more problematic for rectal cancer patients [16, 17]. In relation to stoma, it has been indicated that those who had an ostomy reversal had more bowel issues than those who retained a stoma [18], although these findings were not confirmed in another large scale study [13]. Age has also been reported as influential, with younger survivors experiencing a higher number of symptoms [13, 19, 20].

The results of these studies suggest that although some consensus exists in terms of time since treatment and the influence of age on symptoms in colorectal cancer survivors, existing evidence is limited and information regarding the full extent of symptoms and survivorship is fragmented. To inform survivorship care, it is necessary to identify specific frequently occurring symptoms and influencing factors. It seems the optimum timeframe to investigate this is between one and three years after diagnosis as the specific effect of the early post-operative period is avoided and physical symptoms remain relatively constant at this stage [13, 14, 21]. However, within the medium term survivorship time period, of approximately one to three years, evidence on symptom burden in colorectal cancer survivors is scarce, with just two studies specifically focusing on this [13, 14]. In addition, there is little literature available examining the association of symptoms with each other - only two previous studies considered this with both studies investigating symptoms in long term cancer survivors, and only one focusing specifically on colorectal cancer [22, 23]_._ Addressing this gap could inform future service development and the provision of support for medium term colorectal cancer survivors. Consequently, the aim of this study is to investigate the frequency of symptom occurrence, symptoms that are experienced together, and the relationship of symptoms to demographic and clinical factors in colorectal cancer survivors nine months to three years post-diagnosis.

## Methods

### Design and participants

The study methods have been described in detail elsewhere [24]. Briefly, survivors of primary invasive colorectal cancer diagnosed between October 2007 and September 2009 were identified from the National Cancer Registry Ireland (NCRI) in March 2010, and screened for study eligibility by their treating clinicians. Those on the register were excluded if their managing clinician did not respond or they were unaware of their diagnosis, and if they had poor understanding of English, cognitive impairment, were too ill to participate or had died. Study information leaflets, letters of invitation, informed consent forms and questionnaires were sent to 1273 eligible individuals. Those willing to participate returned a completed questionnaire and informed consent form by post to the NCRI.

### Assessment of demographic, clinical and symptom information

Demographic and clinical data were obtained from Registry records and from questionnaire responses. The assessment tools used to measure symptoms were the European Organisation for Research and Treatment of Cancer Quality of Life Questionnaire – Core 30 (EORTC QLQ-C30) and the European Organisation for Research and Treatment of Cancer Quality of Life Questionnaire – Colorectal 29 (EORTC QLQ-CR29), and these have been published previously [25]. These widely used instruments with proven validity and reliability are recommended to be used in conjunction with each other; they contain items that measure symptom frequency in the previous week that are relevant to those diagnosed with cancer in general and colorectal cancer specifically [26–28].

Responses to the symptom scales (3 subscales and 4 single items on the QLQ-C30; 3 subscales on the CR29, which included urinary frequency, blood and mucous in stool and stool frequency, and 11 single items which included urinary incontinence, dysuria, abdominal pain, buttock pain, bloating, taste, flatulence, faecal incontinence, sore skin, impotence and dyspareunia) were scored using the EORTC guidelines, which involved raw scores being transformed to a linear scale ranging from 0 to 100, with a higher score indicating that symptoms are experienced more frequently [28–30]. According to the scoring procedures, patients that experienced a symptom ‘quite a bit’ or ‘very much’ score ≥ 51 on the relevant subscale, whereas those that score ≤ 50 indicated a symptom was ‘not at all’ present, or there ‘a little’ [28, 30, 31]. In the current study, for the most part, analysis is performed with the symptom scores, but the percentage of participants who scored ≥51 is also examined, in order to indicate the most frequently reported symptoms. In this study, respondents with a symptom score ≥ 51 are described as experiencing a symptom “frequently” or “often” and the symptoms with the highest mean scores are described as the “common” or “commonly-reported” symptoms. When a respondent’s score is ≥51 on two symptoms, it is indicated that these two symptoms “occur together”.

### Statistical methods

The statistical package SPSS v.22 was used for analysis. Means and standard deviations of the symptom score scale data were used to identify the commonly-reported symptoms. Spearman rank correlations between scores for pairs of symptoms were used to identify symptoms which tended to occur together; a priori, a rank correlation of 0.30, as the lower bound was applied for this purpose. Bivariate analyses of the relationships between symptom scores and clinical/demographic variables were based on one-way analysis of variance (ANOVA) and statistically significant results are reported. As an additional measure, corresponding non-parametric analyses using Kruskal-Wallis tests were also carried out, and any additional significant results noted and reported. The choice of ANOVA was made because it gives more readable output (mean scores) than the non-parametric approach (which uses ranks). The main purpose here was to identify which symptoms were associated with which factors; the non-parametric tests were included in order to identify any that were missed by the parametric approach. General linear models were fitted separately for each symptom score, to test for relationships of demographic and clinical variables to symptom scores. Selection of explanatory variables for these general linear models followed a standard search procedure: choice of the initial multi-variable models was based on earlier bivariate results, and statistically non-significant explanatory variables were then removed, one by one, from each multi-variable model, until only significant variables remained. Participants with missing values were omitted from any analyses involving those variables. The 5% level of significance was used in analysis of variance and general linear models, without correction for multiple tests.

## Results

### Response rate and sample size

The total number of participants in the study sample was 496, yielding a response rate of 39%. The proportion of non-responders was significantly higher in the age group ≥75 years, but there were no statistically significant differences between responders and non-responders for gender, tumor site, treatment received, stage at diagnosis or time since diagnosis.

### Demographic and clinical characteristics of responders

The demographic and clinical characteristics of the study participants are summarised in Table 1. The age range of participants was 26–93 years, with mean age (±sd) = 67.4(±11.6). Almost two-thirds were male (63%) and 62% had colon cancer. Slightly more than one in five participants (22%) reported having a stoma at the time of survey completion. Most survivors (86%) had undergone surgical resection of the colon or rectum, with or without chemotherapy or radiotherapy.

**Table 1:** Demographic and clinical characteristics

	All respondents (*n* = 496)^a^	
	n	%
Gender		
Female	186	37.5
Male	310	62.5
Age at survey		
≤ 54 years	69	13.9
55–64 years	130	26.2
65–74 years	177	35.7
≥ 75 years	120	24.2
Marital status		
Single	36	7.4
Married	369	75.6
Separated / widowed	83	17.0
Highest education level		
Primary school	149	30.5
Secondary school	230	47.1
Third level	109	22.4
Site		
Colon	307	61.9
Rectum	189	38.1
Stage at diagnosis		
Stage I	90	18.1
Stage II	141	28.4
Stage III	175	35.3
Stage IV	36	7.3
Unknown/unstaged	54	10.9
Time since diagnosis		
≤ 1 year	186	37.5
1–2 years	236	47.6
2–3 years	74	14.9
Stoma		
Never had stoma	243	51.2
Previous stoma	127	26.7
Current stoma	105	22.1
Treatment^b^		
Surgical resection	427	86.3
Chemotherapy	139	28.1
Radiotherapy	81	16.4

^a^Some percentages calculated using denominator less than stated total due to missing data

^b^Treatment within 1 year of diagnosis

### Symptom frequency scores

Means and standard deviations of the symptom scores are presented in Table 2, and also the percentages of participants experiencing each symptom frequently in the previous week (i.e. responding “quite a bit” or “very much”). The three most common symptoms (each scored as ≥51 by more than 20% of respondents) were fatigue, insomnia and flatulence. Scored as ≥51 by 10–20% of respondents were eight additional symptoms: constipation, diarrhea, bloating, appetite loss, weight worry, dry mouth, sore skin and frequent urination. All other symptoms were scored as ≥51 by less than 10% of study respondents.

**Table 2:** Symptom frequency scores and percentage of subjects experiencing each symptom often

Symptom	Mean (±SD)	Scored > 51 (‘quite a bit’/ ‘very much)
*Fatigue* (*n* = 475)	33 (**±**26)	120 (24.2%)
*Insomnia* (*n* = 470)	28 (**±**30)	109 (22.0%)
*Bowel Function*		
- Constipation (*n* = 468)	17 (**±**27)	57 (11.5%)
- Diarrhoea (*n* = 467)	21 (**±**30)	67 (13.5%)
- Flatulence		
(current stoma) (*n* = 107)	38 (**±**29)	27 (25.5%)
(no stoma) (*n* = 252)	37 (**±**32)	80 (21.6%)
(combined) (*n* = 359)	37 (**±**30)	107 (22.5%)
- Bloating (*n* = 471)	20 (**±**31)	56 (11.3%)
- Blood in stool (n = 471)	3 (**±**27)	5 (1.0%)
- Faecal incontinence		
(current stoma) (*n* = 102)	27 (**±**11)	19 (18.1%)
(no stoma) (*n* = 253)	14 (**±**24)	22 (5.9%)
(combined) (*n* = 355)	18 (**±**27)	41 (8.6%)
- Stool frequency		
(current stoma) (*n* = 106)	17 (**±**24)	8 (7.5%)
(no stoma) (*n* = 254)	24 (**±**25)	25 (6.8%)
(combined) (*n* = 360)	22 (**±**25)	33 (6.9%)
*Nutrition*		
- Appetite loss (*n* = 474)	15 (**±**26)	56 (11.3%)
- Weight worry (n = 467)	22 (**±**27)	66 (13.3%)
- Taste (n = 467)	13 (**±**24)	38 (7.7%)
- Nausea & vomiting (n = 475)	10 (**±**20)	21 (4.2%)
- Dry mouth (n = 471)	17 (**±**27)	59 (11.9%)
*Pain*		
- General pain (n = 474)	19 (**±**25)	47 (9.5%)
- Abdominal pain (*n* = 469)	12 (**±**22)	32 (6.5%)
- Buttock pain (*n* = 466)	14 (**±**25)	45 (9.1%)
*Dermatological Issues*		
- Sore skin		
(current stoma) (*n* = 105)	27 (**±**30)	20 (19.0%)
(no stoma) (n = 253)	18 (**±**27)	31 (8.4%)
(combined) (*n* = 358)	21 (**±**28)	51 (10.7%)
*Urinary Issues*		
- Dysuria (n = 470)	4 (**±**15)	11 (2.2%)
- Urinary frequency (n = 466)	33 (**±**26)	82 (16.5%)
- Urinary incontinence (*n* = 465)	12 (**±**22)	27 (5.4%)

At least one of the eleven most common symptoms was experienced frequently by almost every respondent (98.8%). Nearly two-thirds of respondents (66.3%) experienced at least two of these eleven symptoms together. More than one in seven (15.5%) experienced five or more of these eleven symptoms together.

### Relationships among symptoms

Symptoms for which the rank correlations were 0.3 or more with other symptoms are displayed in Table 3. A high positive correlation between two symptom scores indicate that these symptoms tend to occur (or not) together - both are experienced, or neither are experienced to any great extent.

**Table 3:** Correlations between symptom frequency scores^a^

	*Fatig-ue*	*Insom-nia*	*Const-ipation*	*Diarr-hea*	*Flat-ulence* *(stoma)*	*Flat-* *ulence* *(no stoma)*	*Bloat-* *Ing*	*F.I.* *(stoma)*	*F.I.* *(no stoma)*	*S.F.* *(stoma)*	*S.F.* *(no stoma)*	*Appetite loss*	*Weight worry*	*Taste*	*Nausea* *vomit*	*Dry mouth*	*Gen. pain*	*Abdo pain*	*Butt.* *pain*	*Sore skin* *(stoma)*	*Sore skin* *(no stoma)*
*Fatigue*		.452^**^	.345^**^	.466^**^		.351^**^	.382^**^			.	362**	.564^**^	.323^**^	.353^**^	.559^**^	.373^**^	.612^**^	.334^**^	.316^**^		
*Insomnia*							.305^**^					.324^**^	.305^**^		.353^**^		.354^**^		.317^**^		
*Bowel Function*																					
- Constipation				.327^**^											.331^**^		.349^**^				
- Diarrhoea						.386**	.330^**^		.383^**^		.537^**^	.338^**^			.434^**^		.397^**^				
- Flatulence																					
(current stoma)												.346^**^			.389^**^		.396^**^			.316^**^	
(no stoma)							.369^**^		.469^**^		.468^**^						.326^**^		.369^**^		
- Bloating									.321^**^		.402^**^				312^**^		.335^**^	.378^**^	.347^**^		
- Faecal incontinence																					
(current stoma)										.425^**^								.319^**^	.374^**^	.396^**^	
(no stoma)											.415^**^								.314^**^		.358^**^
- Stool frequency																					
(current stoma)													.319^**^							.364^**^	
(no stoma)															.314^**^		.301^**^		.446^**^		.392^**^
*Nutrition*																					
- Appetite loss														.380^**^	.532^**^	.359^**^	.466^**^				
- Weight worry																					
- Taste																				.416^**^	
- Nausea & vomiting																.340^**^	.507^**^	.349^**^			
- Dry mouth																	.304^**^				
*Pain*																					
- General pain																		.508^**^	.399^**^	.322^**^	
- Abdominal pain																			.384^**^		
- Buttock pain																					503^**^

^**^p ≤ 0.01 *F.I.* Faecal Incontinence, *S.F* Stool Frequency, Gen pain = General pain; Abdo pain = Abdominal pain; Butt pain = Buttock pain

^a^Spearman’s rank correlations. Only those correlations ≥0.30 are displayed

The large number of significant correlations in Table 3 indicates that the range of symptoms which occur together is wide. For example, it shows that fatigue tends to occur together with six of the other ten most common symptoms, and also with another eight of the less commonly-reported symptoms. The highest correlation (0.612) is between fatigue and general pain, the latter being one of the less commonly-reported symptoms; only 47 respondents (9.5%) reported experiencing general pain frequently, but 36 of these 47 (76.6%) also reported frequent fatigue. Similarly, while nausea/vomiting was common in only 21 respondents, 19 of these 21(90.5%) also reported frequent fatigue.

### Relationships between symptom scores, and demographic and clinical variables

Table 4 displays the symptom scores which differ significantly by the demographic (gender and age) and clinical (site, stage and stoma) variables. The *p*-values displayed, except where indicated otherwise, are from analysis of variance.

**Table 4:** Statistically significant relationships between mean symptom frequency scores (+/− sd) and demographic and clinical variables

	Fatigue	Insomnia	Diarrhoea	Flat. *No stoma*	Bloating	F.I. *Stoma*	F.I. *Combined*	S.F. *No stoma*	Appetite Loss	Taste	Nausea Vomit	Dry mouth	Gen pain	Abdo pain	Butt. pain	Sore skin *No stoma*	Sore skin *Combined*	U.F.	U.I.
Gender																			
*Male*		26.29^*^ (±30.32)			17.56^*^ (±25.97)				13.40^*^ (±24.97)					10.70^a^ (±20.59)				34.47^*^ (±25.88)	
*Female*		32.56^*^ (±30.37)			23.12^*^ (±28.16)				18.88^*^ (±24.97)					14.45^a^ (±23.08)				29.36^*^ (±25.08)	
Age																			
*≤ 54*					25.49^a^ (±25.18)			33.33^*^ (±27.22)						21.89^***^ (±23.61)	23.38^***^ (±28.44)		24.56^a^ (±29.23)	27.61^**^ (±25.39)	
*55–64*					20.73^a^ (±29.07)			21.72^*^ (±23.93)						15.47^***^ (±26.28)	14.81^***^ (±27.18)		24.74^a^ (±29.77)	28.31^**^ (±25.76)	
*≥ 65*					17.63^a^ (±26.11)			22.42^*^ (±23.66)						8.18^***^ (±17.41)	10.50^***^ (±22.04)		17.65^a^ (±26.35)	35.78^**^ (±25.33)	
Site																			
*Colon*			17.13^**^ (±27.54)	31.08^***^ (±31.67)		8.38^***^ (±18.65)		34.98^**^ (±22.22)			8.70^a^ (±19.73)		16.72^**^ (±24.85)	9.72^**^ (±19.63)	9.24^***^ (±21.59)	13.70^***^ (±23.05)			8.51^***^ (±18.77)
*Rectum*			26.82^**^ (±32.97)	47.19^***^ (±30.90)		24.44^***^ (±29.48)		18.20^**^ (±20.94)			10.99^a^ (±19.86)		22.62^**^ (±25.92)	15.84^**^ (±19.63)	20.26^***^ (±28.02)	25.93^***^ (±30.71)			16.39^***^ (±25.81)
Stage																			
*I*					13.73^*^ (±21.99)	7.84^*^ (±14.58)													
*II*					17.78^*^ (±26.64)	29.49^*^ (±30.30)													
*III*					20.86^*^ (±27.49)	25.44^*^ (±27.33)													
*IV*					27.62^*^ (±27.40)	38.89^*^ (±38.97)													
Stoma																			
*Previous*	31.81^**^ (± 24.35)	31.71^**^ (±31.03)	24.66^*^ (±31.32)				19.94^***^ (±25.44)		14.63^*^ (± 24.94)	10.38^***^ (±20.59)	9.20^*^ (±19.68)		21.07^***^ (±25.50)	13.71^*^ (±24.05)	20.16^***^ (±27.81)		22.64^***^ (±27.81)		9.29^**^ (±16.74)
*Current*	41.83^**^ (±26.13)	35.64^**^ (±34.72)	24.33^*^ (±29.21)				25.18^***^ (±28.79)		22.55^*^ (±30.82)	22.22^***^ (±32.27)	22.21^*^ (±14.54)		28.38^***^ (±30.24)	16.33^*^ (±23.92)	15.84^***^ (±26.50)		27.78^***^ (±29.69)		18.37^**^ (±29.55)
*Never*	29.80^**^ (±25.49)	24.60^**^ (±27.77)	17.03^*^ (±28.80)	33.33^a^ (±33.44)			11.86^***^ (±25.13)		12.50^*^ (±26.19)	9.40^***^ (±19.57)	7.86^*^ (±18.89)		14.49^***^ (±22.36)	9.65^*^ (±19.15)	8.78^***^ (±20.88)		14.89^***^ (±25.77)		10.38^**^ (±20.86)

*Flat* Flatulence, *F.I* Faecal incontinence, *S.F* Stool frequency, *Gen. pain* General pain, *Abdo. pain* Abdominal pain, *Butt. pain* Buttock pain, *U.F* Urinary frequency, *U.I* Urinary incontinence

^*^p ≤ 0.05; ^**^p ≤ 0.01; ^***^p ≤ 0.001

^a^= Statistical significance demonstrated in nonparametric test only

Symptom scores did not differ significantly across the six possible treatment categories (Surgery Only; Chemotherapy Only; Radiotherapy Only; Surgery Chemotherapy & Radiotherapy; Surgery & Chemotherapy; Surgery & Radiotherapy) but this finding is tentative because of the very small numbers in some treatment categories. When treatment was considered as binomial variables (cancer-directed surgery yes/no, chemotherapy yes/no and radiotherapy yes/no) just a small number of significant relationships were identified; these included flatulence (higher in those with a stoma who had chemotherapy, *p* = 0.03), and abdominal pain (higher in those that received radiotherapy, *p* = 0.02).

The findings from the multivariable linear models for the eight symptoms that demonstrated statistical significance based on bivariate results are summarized in Table 5. More detailed information (model coefficients, confidence intervals for these coefficients, and r-squared values for each model), is displayed in Additional file 1: Table S1. The r-squared values are low for all eight models, ranging from 0.02, for the model relating urinary frequency to age, up to 0.08, for the relationship of buttock pain to age, site and stoma. Variation in these demographic and clinical variables, therefore, explains only a small proportion of variation in these symptom scores. Stoma is significantly related to five of the eight symptoms. Site is related to four symptoms, age is associated with three symptoms and gender is related to two symptoms.

**Table 5:** Summary of results from multivariable models: statistically significant relationships between higher symptom scores and multiple demographic/clinical variables^a^

Symptom	Bivariate Relationships	Multivariate Relationships
*Abdominal pain*	≤54 years Current stoma Female gender Rectal cancer	≤54 years Rectal cancer
*Insomnia*	Female gender Current stoma	Female gender Current stoma
*Diarrhoea*	Rectal cancer Previous stoma	Rectal cancer
*Appetite loss*	Female gender Current stoma	Female gender Current stoma
*Buttock pain*	≤54 years Rectal cancer Previous stoma	≤54 years Rectal cancer Previous stoma
*Sore skin*	55–64 years Current stoma	Current stoma
*Urinary frequency*	Male gender ≥65 years	≥65 years
*Urinary incontinence*	Rectal cancer Current stoma	Rectal cancer Current stoma

^a^There were no other significant bivariate or multivariate relationships for all other symptoms

## Discussion

In this study, symptom frequency, symptoms that occur together, and the clinical and demographic variables associated with such symptoms were investigated in colorectal cancer survivors nine months to three years post diagnosis. The results indicate that three symptoms - fatigue, insomnia and flatulence - remain frequent for some individuals nine months to three years following diagnosis. Moreover, it has been demonstrated that in colorectal cancer survivors nine months to three years post diagnosis, symptoms tend to occur together rather than in isolation. Additionally, in relation to clinical variables, those with a stoma reported higher scores for several symptoms. Consequently, it is evident that symptom burden exists for groups of individuals, which is often multidimensional in nature.

Regarding symptom presentation, fatigue, insomnia and flatulence, were scored as ≥51 on a scale of 0–100 in more than 20% of the study participants. The findings in relation to fatigue and insomnia are also reflected in previous studies that investigated these symptoms in colorectal cancer survivors [12, 13, 15]. Results of a cross-sectional study that examined symptoms and quality of life in colorectal cancer survivors within a similar timeframe of post diagnosis indicated that 40% of the sample had trouble sleeping and felt tired [13]. That study had a larger sample size and higher response rate than the current study and the two studies used different instruments to assess sleep problems. Furthermore, in that study 20% of survivors reported having little or no appetite [13] which supports the findings of the current investigation where 10% to 20% of respondents scored ≥51 for the symptoms of appetite loss, weight worry and dry mouth. The two studies reported similar prevalence of frequent urination (17% here vs 13% by Downing et al. (2015)) [13]. Interestingly, when compared with the general population, research in the United States has indicated that older survivors of colorectal cancer reported similar levels of urinary issues [32], thus demonstrating that other variables may be influential.

Therefore, in order to gain a more comprehensive view, it is necessary to consider the effect of other variables on symptom scores, thus warranting further examination of the association of symptoms with one another. As well as being the most common symptoms, fatigue, insomnia and flatulence, tended to occur together, and with many other symptoms. Of particular note were the associations between fatigue, insomnia, bloating, nausea and vomiting and general pain. In addition, fatigue also correlated with diarrhea, weight worry, taste, dry mouth, abdominal and buttock pain, flatulence and stool frequency. Consequently, the results of this current investigation strongly indicate that symptoms do not occur in isolation. A recent study that examined symptom clusters in colorectal cancer survivors ≤ 2 years and ≥ 10 years post diagnosis confirms this, with anxiety, fatigue and depression clustering, in addition to pain and insomnia [23]. However, in that study only five other symptoms in addition to fatigue were analyzed [23]. Therefore, the current investigation offers a more comprehensive view and demonstrates that although symptom scores may be higher for certain symptoms, in order to optimize survivorship care, these cannot be considered as singular issues.

In relation to the influence of clinical and demographic variables on symptoms, results of the current study should be interpreted with caution as, although there were a range of statistically significant relationships between clinical and demographic variables and symptoms, following multivariate analysis, these relationships were generally weak, with more than 90% of variance of symptom scores remaining unexplained by such variables. In terms of understanding this variance in symptom scores further, this may be explained by examining other variables that were not analyzed here, such as co-morbidities and psychological distress, both of which have been previously identified as influential [23]. Therefore, when findings of the current investigation are examined in isolation, this suggests that the clinical and socio-demographic data are of limited use in helping target supportive care interventions. However, the results from the analysis of these variables in this study are supported by findings of previous investigations.

Regarding presence of a stoma, this was identified as the single most common factor associated with increased symptom scores. Interestingly, Downing et al. (2015) indicated that the presence of a stoma significantly reduced the proportion of respondents reporting ‘perfect’ health when compared with those that had no stoma (19% versus 40%) [13]. Existing literature in relation to the influence of site on symptoms also supports the findings of the current study, in that survivors of rectal cancer report higher symptom scores than those diagnosed with colon cancer [13, 16, 17]. In addition, consensus also exists in terms of age and the presence of symptoms associated with colorectal cancer survivorship, with more problems reported by younger respondents [13, 14]. It has been suggested that the influence of age on the burden of disease may explain these findings, as older adults may assess their physical health in terms of their peers, rather than the ideal of perfect health [14]. There was also little or no statistical evidence of a relationship between symptom scores and stage of cancer, type of cancer treatment, or time since diagnosis. Regarding treatment, results of the current investigation agree with previous studies of longer term survivors [9, 18]. Existing literature that examined the influence of time since diagnosis on symptoms is limited to just a single study within the one to three year time frame; this reports similar findings to the current investigation, with only modest improvements noted from the first to the third year after diagnosis. The study which has been discussed previously, that also identified symptom clusters in colorectal cancer survivors, albeit over a longer time frame post diagnosis (≤ 2 years and ≥ 10 years) similarly reported that time since diagnosis demonstrated little influence on symptom burden [23].

There are a number of limitations in relation to the current investigation that require consideration. Specifically in relation to surgical resection of the colon or rectum, results of this study are slightly higher than rates reported by Carsin et al. (2008) [33] on colorectal cancer treatment patterns in Ireland for this time (86% versus 78%). However, it must be noted that endoscopic surgery for removal of early stage tumors was not recorded and so there may be incomplete surgery data for the remaining 14% of the sample investigated which must be considered, as the type of surgery should not be neglected as a potential influencing factor on symptom presentation. In addition, it is not possible to disentangle colorectal cancer specific symptoms and general symptoms due to the lack of a non-cancer control group as well as information regarding comorbidities. Also, the low overall response rate of 39% and a significant proportion of non-responders in the age category ≥75 years may have led to an imprecise estimation of symptom scores in this study. The actual number of symptoms experienced frequently, and experienced together, may be even higher than reported here. Clinically, symptoms present nine months to three years post diagnosis should not, therefore, be considered in isolation. This may inform future colorectal cancer survivorship care.

The major recommendation for practice that follows from these findings is that there should be thorough assessment of the presence of symptoms in the follow-up of medium term colorectal cancer survivors, in order to inform tailored patient-centred support and care. There needs to be heightened awareness among the clinical team, that symptoms tend to be multi-dimensional in nature in this patient group and that those patients with a stoma may be more likely to need support. In addition, particular attention should be paid to assessing and providing support around fatigue, insomnia and flatulence.

## Conclusion

This study identified that fatigue, insomnia and flatulence were the most common symptoms in colorectal cancer survivors nine months to three years post their diagnosis, and that these symptoms, and many others, tended to occur together. In addition, presence of a current stoma was the single most common factor associated with increased symptom scores, although this relationship was weak, despite reaching statistical significance. These findings may inform clinical practice as the identification, implementation and evaluation of targeted interventions during the nine month to three year post diagnosis timeframe would enable supported self-management of problematic symptoms such as fatigue, insomnia and flatulence, particularly in relation to those with a stoma. In addition, research exploring the influence of symptoms on quality of life and functioning is required in order to gain a more comprehensive view of the consequences of symptom burden.

## Additional file

Additional file 1: **Table S1**. Fitted main effect general linear models for the relationship of symptom scores to demographic/clinical variables. The findings from the multivariable linear models for the eight symptoms that demonstrated statistical significance based on bivariate results are summarized in Table 5. More detailed information (model coefficients, confidence intervals for these coefficients, and r-squared values for each model), is displayed in Table S1. (DOCX 30 kb)

## Abbreviations

CREW: ColoREctal Wellbeing
EORTC QLQ-C30: European Organisation for Research and Treatment of Cancer Quality of Life Questionnaire – Core 30
EORTC QLQ-CR29: European Organisation for Research and Treatment of Cancer Quality of Life Questionnaire – Colorectal 29
NCRI: National Cancer Registry Ireland
