# Supplementary material for: Colorectal cancer survivors: an investigation of symptom burden and influencing factors
Source: BMC Cancer. 2018 Oct 22;18:1022. doi: 10.1186/s12885-018-4923-3 (PMC6198486; doi:10.1186/s12885-018-4923-3)
Supplement: Supplementary file 1 — Table S1. Fitted main effect general linear models for the relationship of symptom scores to demographic/clinical variables. The findings from the multivariable linear models for the eight symptoms that demonstrated statistical significance based on bivariate results are summarized in Table 5. More detailed information (model coefficients, confidence intervals for these coefficients, and r-squared values for each model), is displayed in Table S1. (DOCX 30 kb) [file 12885_2018_4923_MOESM1_ESM.docx]

**Table S1: Fitted main effect general linear models for the relationship of symptom scores to demographic/clinical variables**

| **Abdominal Pain *(r-sq = 0.07)*** | **Age** | | | **Site** | | **Stoma** | | | **Gender** | |
| --- | --- | --- | --- | --- | --- | --- | --- | --- | --- | --- |
|  | *≤ 55years* | *55-64years* | *≥ 65years* | *Rectum* | *Colon* | *Previous* | *Current* | *Never* | *Male* | *Female* |
| *Intercept (95% CI Lower Bound – Upper Bound)* | 6.20 (3.33 - 9.06) | | | | |  |  |  |  |  |
| *Coefficient* | 11.89 | 7.63 | 0 | 5.39 | 0 |  |  |  |  |  |
| *95% CI Lower Bound*  *Upper Bound* | 6.50  17.27 | 3.10  12.16 |  | 1.47  9.32 |  |  |  |  |  |  |
|  |  | | |  | |  | | |  | |
| **Buttock Pain *(r-sq = 0.08)*** |  |  |  |  |  |  |  |  |  |  |
| *Intercept (95% CI Lower Bound – Upper Bound)* | 5.20 (1.53 – 8.86) | | | | | | | |  |  |
| *Coefficient* | 8.83 | 4.74 | 0 | 6.88 | 0 | 8.02 | 3.86 | 0 |  |  |
| *95% CI Lower Bound*  *Upper Bound* | 2.44  15.23 | -0.54  10.03 |  | 1.82  11.93 |  | 2.40  13.64 | -2.21  9.92 |  |  |  |
|  |  | | |  | |  | | |  | |
| **Urinary Frequency *(r-sq = 0.02)*** |  |  |  |  |  |  |  |  |  |  |
| *Intercept (95% CI Lower Bound – Upper Bound)* | 35.78 (32.75 – 38.80) | | |  |  |  |  |  |  |  |
| *Coefficient* | -8.22 | -7.39 | 0 |  |  |  |  |  |  |  |
| *95% CI Lower Bound*  *Upper Bound* | -14.74  -1.69 | -12.90  -1.88 |  |  |  |  |  |  |  |  |
|  |  | | |  | |  | | |  | |
| **Insomnia *(r-sq = 0.04)*** |  |  |  |  |  |  |  |  |  |  |
| *Intercept (95% CI Lower Bound – Upper Bound)* |  |  |  |  |  | 21.43 (16.80 – 26.07) | | | | |
| *Coefficient* |  |  |  |  |  | 7.63 | 12.31 | 0 | 0 | 7.41 |
| *95% CI Lower Bound*  *Upper Bound* |  |  |  |  |  | 0.99  14.27 | 5.16  19.45 |  |  | 1.58  13.23 |
|  |  | | |  | |  | | |  | |
| **Appetite Loss *(r-sq = 0.04)*** |  |  |  |  |  |  |  |  |  |  |
| *Intercept (95% CI Lower Bound – Upper Bound)* |  |  |  |  |  | 9.64 (5.70 – 13.58) | | | | |
| *Coefficient* |  |  |  |  |  | 2.60 | 11.20 | 0 |  | 6.70 |
| *95% CI Lower Bound*  *Upper Bound* |  |  |  |  |  | -3.06  8.26 | 5.12  17.28 |  |  | 1.74  11.65 |
|  |  | | |  | |  | | |  | |
| **Diarrhoea *(r-sq = 0.03)*** |  |  |  |  |  |  |  |  |  |  |
| *Intercept (95% CI Lower Bound – Upper Bound)* |  |  |  | 17.13 (13.69 – 20.57 | |  |  |  |  |  |
| *Coefficient* |  |  |  | 9.69 | 0 |  |  |  |  |  |
| *95% CI Lower Bound*  *Upper Bound* |  |  |  | 4.12  15.25 |  |  |  |  |  |  |
|  |  | | |  | |  | | |  | |
| **Urinary Incontinence *(r-sq = 0.05)*** |  |  |  |  |  |  |  |  |  |  |
| *Intercept (95% CI Lower Bound – Upper Bound)* |  |  |  | 8.88 (5.91 – 11.85) | | | | |  |  |
| *Coefficient* |  |  |  | 7.97 | 0 | -4.16 | 4.61 | 0 |  |  |
| *95% CI Lower Bound*  *Upper Bound* |  |  |  | 3.39  12.55 |  | -9.29  0.97 | -0.93  10.15 |  |  |  |
|  |  | | |  | |  | | |  | |
| **Sore Skin *(r-sq = 0.04)*** |  |  |  |  |  |  |  |  |  |  |
| *Intercept (95% CI Lower Bound – Upper Bound)* |  |  |  |  |  | 14.89 (10.47 – 19.31) | | |  |  |
| *Coefficient* |  |  |  |  |  | 7.75 | 12.89 | 0 |  |  |
| *95% CI Lower Bound*  *Upper Bound* |  |  |  |  |  | 0.89  14.62 | 5.82  19.96 |  |  |  |

CI = Confidence Interval
